# The Burden of Early Childhood Caries in Children under 5 Years Old in the European Union and Associated Risk Factors: An Ecological Study

**DOI:** 10.3390/nu13020455

**Published:** 2021-01-29

**Authors:** Zsuzsa Bencze, Nour Mahrouseh, Carlos Alexandre Soares Andrade, Nóra Kovács, Orsolya Varga

**Affiliations:** Department of Public Health and Epidemiology, Faculty of Medicine, University of Debrecen, 4032 Debrecen, Hungary; bencze.zsuzsa@sph.unideb.hu (Z.B.); nour.mahrouseh@med.unideb.hu (N.M.); soares.andrade@med.unideb.hu (C.A.S.A.); kovacs.nora@med.unideb.hu (N.K.)

**Keywords:** early childhood caries (ECC), disease burden, added sugar, global burden of disease database, oral health, informing policy, socioeconomic inequalities, sugar consumption

## Abstract

The associations among early childhood caries (ECC), socioeconomic status, and sugar consumption are of the utmost importance, due to their potential policy implications. The purpose of this study was to identify trends in ECC burden in children under 5 years old among European Union (EU) member states over time and to evaluate the relationship with its risk factors. Global Burden of Disease 2019 data were analyzed to estimate the burden of ECC over time, specifically incidence, prevalence, and years lived with disability (YLDs) for children under 5 years old. Four ecological variables with a potential effect on YLDs for ECC were used to investigate the association between 2014 and 2017. The YLDs rate was consistently higher among Eastern EU countries over time. Univariate models showed a positive significant association between at-risk-of-poverty rate and YLDs rate, while GDP per capita and urbanization were inversely associated with YLDs rate. In the multivariate analysis, sugar consumption, GDP per capita and urbanization showed significant association with YLDs rate. After stratification by region, association remained significant only in the Eastern EU countries between GDP, urbanization, and YLDs rate, while sugar consumption and at-risk-of-poverty rate had no significant impact on YLDs rates. This study found increasing ECC burden in the EU. The complexity of the problem indicates the need for innovative and personalized policy approaches to tackle the disease.

## 1. Introduction

Early childhood caries (ECC) is a disease of primary teeth among children under 6 years of age [1]. ECC represents a major public health issue among children worldwide and has become prevalent, especially in low- and middle-income countries due to changes in lifestyle and the regular consumption of cariogenic foods [2]. ECC is often left untreated, causing painful conditions and impairing children’s general health, growth, and development. Furthermore, if hospitalization or surgical procedures are necessary, it can affect the whole family’s life [3]. Oral health is part of systemic health, and the complications of caries in childhood can cause systemic problems as well [4].

ECC is a multifactorial disease [5,6]. Risk factors include microbiological, dietary, and environmental factors [7,8,9,10]. The impact of socioeconomic indicators, such as socioeconomic status, education, income [11], and sugar consumption, are well studied, indicating evidence of their association. According to a Swedish multilevel analysis, socioeconomic status is a stronger indicator for the risk of dental caries experience than gender or age [6,12]. The mother’s education level and low income are found to strongly correlate with higher ECC experience [6,13]. Sugar-rich food and added sugar have received much attention among dietary risk factors for the development of dental caries in children [14]. Higher sugar-sweetened beverage intake is associated with more severe ECC [15]. However, not only does the quantity of sugar intake have an effect on oral health in early childhood but also the frequency of sugar intake, infant feeding patterns, and nutritional habits (e.g., night bottles) [16]. The WHO emphasizes the role of good nutrition and a balanced diet in early childhood and recommends the avoidance of free sugars for caries reduction [17]. Although sugar is definitely cariogenic, opinions are divided on the strength of the causal link regarding only the sugar itself and ECC [18]. Surveillance is recommended by the WHO to facilitate ECC prevention and to assess interventions [2]. The significance of early detection and the prevention of ECC has been emphasized by several studies [6,19,20]. In a US-based study, a strong association was described between sugar intake and caries experience and also between added sugar intake and social background, such as socioeconomic disadvantage or level of urbanization [21]. The area characteristics, such as sugary food accessibility through local stores, also have an impact on the prevalence of ECC [22].

Since risk factors for children with lower socioeconomic background are different, the applied preventive measures should address these special needs [23]. In the European Union (EU), sugar taxation has proved its efficacy in reducing dental caries [24]. Prevention programs are available for high-risk groups (dental and dietary education of caregivers in infancy, fluoride application, milk programs for preschool children) and have proven successful, but are applied only in a limited number of European countries [25]. Therefore, trends of ECC and its burden are different between European member states. Due to the complex causal relations of the disease, the analysis should reflect not only oral health status but also the social environment [26,27].

### Aims

The purpose of this study was to identify trends in the EU regarding the burden of ECC in children under 5 years old and to evaluate the level of association between the burden of ECC and its risk factors over time.

## 2. Materials and Methods

### 2.1. Data

Three public databases were used to gather data on ECC and related factors for this study. Data on the burden of caries of primary teeth (for the under 5 years age group) were accessed through the Global Health Data Exchange database [28], while socioeconomic data for EU member states were from EUROSTAT [29], and data on per capita sugar consumption were obtained from the Food and Agriculture Organization of the United Nations (FAO) [30].

Global Burden of Disease (GBD) provides global, regional, national and, in some cases, subnational estimates of the burden of diseases, injuries, and risk factors. The data are available for download on the GHDx website (http://ghdx.healthdata.org/gbd-results-tool). The Global Burden of Disease Study 2019 (GBD 2019), with recent updates for several types of input data, gives an estimation of, among other oral diseases, caries of primary teeth (this term is referred to in the database as “deciduous teeth”). The major sources of data for the GBD 2019 for caries of primary teeth, among nonfatal health outcomes, are scientific literature and surveys [31]. After surveys and literature data are collected, the prevalence on current decay and incidence are extracted and calculated, creating a database. Then, with the help of the covariates’ database and further analysis, prevalence and incidence by location/year/age/sex for primary teeth caries are defined and count towards severity analysis. Additionally, from scientific literature data, a meta-analysis is carried out to define the duration of pain with patients having caries of primary teeth [32]. The disease burden data are annually available at the international level from 1990 to 2019. The relevant data on the burden of caries of primary teeth are expressed in terms of incidence, prevalence, years lived with disability (YLDs), and age-standardized YLDs. Health indicators, as defined by the GBD, are as follows: incidence, meaning the number of new cases of a given cause during a given period in a specified population; prevalence, which is the proportion of people in a population who comprise a case of a disease, injury, or sequela; YLDs, which means the years lived with any short-term or long-term health loss, weighted for severity by the disability weights. Cause-specific YLDs are calculated by multiplying sequela-level prevalence with corresponding disability weights, which were taken from population and internet surveys consisting of more than 60,000 persons and adjusted for comorbidity through microsimulation [33]. The use of YLDs is crucial to express the burden of nonfatal health loss. Mortality directly associated with ECC is not considered in the literature. Data are available by gender, age, location, and year and are presented as number, percent, or rate. The selected age group for analysis was under 5 years of age. Although ECC is a disease of primary teeth among children under 6 years of age [1], since ECC is most prevalent in early childhood and, considering the limited age groups provided by GBD, the under age 5 group selection was the most appropriate for this analysis to present the burden of caries of primary teeth.

The EUROSTAT database provides European Union statistics and has various data sources, including national reports and surveys. Regarding this study, the European Union Statistics on Income and Living Conditions (EU-SILC) has a special relevance as it has provided comparable cross-country data on income, poverty, social exclusion, and living conditions on a yearly basis since 2003. We selected the relevant databases related to economic, population, and social conditions to access indicators of inequality and socioeconomic risk factors for ECC—the proportion of people at risk of poverty (calculated as the share of persons with an equivalized disposable income below the risk-of-poverty threshold), GDP per capita in purchasing power standards (PPS, which is expressed in relation to the European Union average and allows meaningful volume comparisons of GDP between countries, eliminating the differences in price levels), and the proportion of population living in cities for urbanization data (distribution of population by degree of urbanization).

The FAO Food Balance Sheet presents a comprehensive picture of the pattern of a country’s food supply during a specified reference period for several food modules. Data related to sugar and sweeteners are available for between 2014 and 2017 due to the methodological changes in data processing in 2013 and level of data availability until 2017. The key difference between the current and previous food balance methodologies is the absence of a balancer variable. As there is no international database available to provide explicit data on national sugar consumption, the food supply data on food availability are widely used to estimate sugar consumption. The food balance items, selected for analysis, were the aggregated items of sugars and sweeteners. These include data of the types of sugar (beet, cane, raw, refined, centrifugal and non-centrifugal, confectionery, or flavored) and also other types of sweeteners, such as fructose, maltose, glucose, maple sugar, syrups, molasses, lactose, and non-alcoholic beverages.

### 2.2. Study Design

The results are presented in two parts. Part 1 contains data on the burden of early childhood caries in the European Union Member States from 1990 to 2019; part 2 presents the changes over time in YLDs rate of early childhood caries and its potential risk factors (sugar consumption, at risk of poverty, GDP and degree of urbanization) from 2014 to 2019. Due to limited data on sugar consumption, the regression models were performed for the time interval from 2014 to 2017.

In the first part of the study, we wanted to get a comprehensive picture of the burden of early childhood caries for the 28 EU member states. Accordingly, the burden of disease of caries of primary teeth among children aged under five years was estimated using the GBD database. We included available YLDs data between 1990 and 2019 for the analysis to provide an overview of the ECC burden shift over time. Furthermore, the data of 2019 were analyzed to estimate the current burden in detail in the EU region using incidence, prevalence, and YLDs rate measures among children aged under 5 years old, as well as age-standardized YLDs. Since many useful observations emerge from addressing differences in gender in ecological studies, and since this can standardize potential confounders [34], gender was part of the analysis.

In the second part of the study, means (SD) of the ecological variables of countries were calculated by years in the descriptive statistics. Means per year were presented to visualize how variables changed over time. Means from 2014 and 2019 were compared, and absolute differences and relative differences (calculated as absolute difference relative to mean from 2014) were calculated.

The effect of four ecological variables were assessed in relation to YLDs rate. The YLDs rate for early childhood caries of primary teeth among children aged under 5 years old was the dependent variable in the analyses. The independent variables were selected as sugar consumption (kg/capita/year), the proportion of people at risk of poverty (%), GDP per capita (in purchasing power standards (PPS)), and the proportion of population living in cities (degree of urbanization (%)) [34,35]. Due to the limited data availability for sugar consumption, analyses were performed for a 4-year time interval (from 2014 to 2017).

### 2.3. Statistical Analysis

In the descriptive statistics, changes in dependent and independent variables over time (absolute and relative differences from 2014 to 2019) were calculated, and differences were assessed with two-sample *t*-tests.

Univariate and multivariate linear regression analyses for panel data modeling were performed to examine the effect of factors that might be linked to inequality for the YLDs rate of ECC over time (2014 to 2017). The model fit was assessed with Akaike’s information criterion and the Bayesian information criterion, showing that the panel data model fits the data well. The number of observations in the regression models was based on the countries multiplied by each year. Considering the potential regional differences between the Eastern and Western countries of the EU, a stratified analysis for the two geographical regions was conducted. During the division, Eastern countries included Bulgaria, Croatia, Czechia, Estonia, Hungary, Latvia, Lithuania, Poland, Romania, Slovakia, and Slovenia; Western countries covered Austria, Belgium, Cyprus, Denmark, Finland, France, Germany, Greece, Ireland, Italy, Luxembourg, Malta, Netherlands, Portugal, Spain, Sweden, and the United Kingdom.

Coefficients with the corresponding 95% confidence intervals (CIs) are presented. Statistical significance was considered as *p* < 0.05. Statistical analyses were performed using STATA IC version 13.0 software.

## 3. Results

### 3.1. Burden of Early Childhood Caries in the European Union Member States

To obtain an overview on the current burden of dental caries of primary teeth, we analyzed data of the countries’ disease burden estimation (for male, female, and both) for the year of 2019 (Supplementary Table S1).

In the European Union, the average incidence rate of dental caries of primary teeth for children under 5 years old was 38,818 per 100,000, with a prevalence of 37.2% and a YLDs rate of 9.8 per 100,000 in 2019. 

The incidence rate for 2019 (per 100,000 population) was the highest males, females, and both sexes in Poland (53,388). The lowest incidence rate was reported for females (15,261), both sexes (15,767), and males (16,248) in the United Kingdom. The remaining countries have an incidence rate of between 30,000 and 50,000. 

Regarding prevalence, boys in Lithuania and Latvia (57.8%) and Poland (57.2%) had the highest values. The lowest prevalence percent data are under 20%, with the lowest values from UK females (16.8%), UK both sexes (19.1%), and Denmark females (19.3%).

The highest YLDs rate values were from Romania for female and both (17.1) and male (17.0), while the lowest numbers were found in the UK: female (4.5), both (4.7), and male (4.9). 

**Figure 1:**
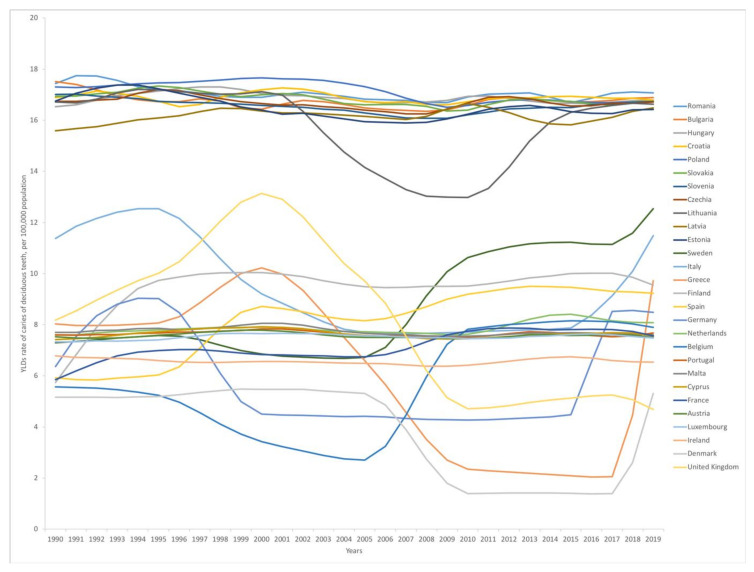
YLDs rate of caries of primary teeth per 100,000 population between 1990 and 2019 (presented age group: under 5 years of age). Source: Global Burden of Disease database. YLDs: years lived with disability.

YLDs rate for primary teeth for the under 5 age group was also examined from 1990 to 2019 to present the disease burden over time, as seen in Figure 1. Two principal groups are distinguished: group 1, with a constantly higher YLDs rate; group 2, with a lower YLDs rate with periodical increases and decreases. Bulgaria, Croatia, Czechia, Estonia, Hungary, Latvia, Lithuania, Poland, Romania, Slovakia, and Slovenia show a constant trend of higher values over the examined years. In Lithuania, there is a decreasing curve between 2002 and 2016, whereas the values remain high for the remaining periods. In Belgium and Sweden, there is a sharp increase, starting in 2005 and 2006. France and Spain also show a slight increase in the values around 2006 to 2007. From 2015 to 2017, there is an increasing trend in YLDs rate in Germany and Italy (from 2015) and Denmark, Greece, and Sweden (from 2017).

The YLDs rate difference (1990 vs. 2019) has increased in Sweden (5.2), Finland (3.8), Spain (3.3), Belgium (2.3), and Germany (2.1). However, even considering this increase, none of these countries reached the YLDs rate of the group 1 countries (see Figure 1).

### 3.2. Association between YLDs Rate of Early Childhood Caries and Its Risk Factors

We aimed to analyze the association between YLDs rate and ECC risk factors. The data on ECC risk factors were available mainly from 2014 to 2019. For this reason, we investigated the changes over time in the 2014 to 2019 period. The change in investigated factors over the period in EU member states (N = 28) is presented in Table 1. The mean (SD) YLDs rate per 100,000 was 11.05 (4.86) during the time interval and increased by 6.6% from 2014 to 2019. Sugar consumption and GDP per capita also increased. A decrease in proportion of people at risk of poverty, degree of urbanization, and proportion of population aged under 5 years was observed. However, none of the observed changes were significant.

The results of panel data regression analyses are presented in Table 2. The analyses were performed for the time interval from 2014 to 2017. Univariate models showed a positive significant association of at-risk-of-poverty rate (coeff. = 0.270; 95% CI: 0.038, 0.501; *p* = 0.023) and YLDs rate, while GDP per capita (coeff. = −0.058; 95% CI: −0.077, -0.039; *p* < 0.001) and urbanization (coeff. = −0.100; 95% CI: −0.163, −0.037; *p* = 0.002) were inversely associated with YLDs rate. In the multivariate model, the sugar consumption (coeff. = 0.104; 95% CI: 0.072, 0.136; *p* < 0.001) and GDP per capita (coeff. = −0.113; 95% CI: −0.134, −0.092; *p* < 0.001) and urbanization (coeff. = −0.151; 95% CI: −0.195, −0.107; *p* < 0.001) showed a significant association with YLDs. Sugar consumption had a positive and independent effect over time in the multivariate model. Results from regression suggest that increases by one unit (kg/capita/year) in the sugar consumption will increase YLDs rate by 0.1 years. The GDP per capita in PPS was associated with a decrease in YLDs rate of 0.11 years, while a one-percent increase in the degree of urbanization was associated with a reduction in YLDs rate by 0.15 years. Interestingly, poverty had no significant effect on YLDs rate (coeff. = 0.023; 95% CI: −0.146, 0.192; *p* = 0.79) in multivariate analysis. The GDP per capita and urbanization show practical importance because the effect remained significant even in the multivariable model.

After stratification by region in the multivariate model, the association between sugar consumption, at-risk-of-poverty rate and YLDs rate was not significant in any strata. However, stratified analysis suggested differences between the two geographical regions in relation to GDP (coeff. = −0.011; 95% CI: −0.020, −0.003; *p* = 0.011) and urbanization (coeff. = −0.021; 95% CI: −0.031, −0.011; *p* < 0.001), the impacts of which on YLDs rate were lower but remained significant in the Eastern countries of the EU. 

## 4. Discussion

The major findings of our study demonstrate an overall increasing burden of ECC in the member states of the EU and only moderate association with either sugar consumption or socioeconomic factors. 

Inequalities are perceptible within and between the EU countries; the burden of disease for primary teeth among the 0–5-year-old population shows a large variety. Eastern European countries have shown higher rates of ECC when compared to more economically developed countries, and our findings are in line with this [36]. The 11 countries with the highest values are above 16.4 YLDs (from highest values): Romania, Bulgaria, Hungary, Croatia, Poland, Slovakia, Slovenia, Czechia, Lithuania, Latvia, and Estonia. These post-Soviet countries joined the EU after 2004. Among countries that joined after 2004, Malta and Cyprus show lower YLDs rates than the other countries. The YLDs rate is between 5 and 8 in Western European countries. A major dental caries rate goal defined by the WHO for the year 2020 is to keep at least 80% of children aged 5 to 6 years old caries-free, a goal that has not been reached by the majority of European countries, especially in the Eastern countries of the EU [36].

These disparities are mostly driven by socioeconomic factors and could be ameliorated by appropriate health policies. Disparities have a crucial impact on people’s health. People affected by lower socioeconomic status represent a vulnerable population within countries, and the average scores for disease burden might not be representative for them. Immigrants, especially involuntary immigrants, are considered vulnerable, with special needs regarding healthcare and dental care. According to a German study, immigrants suffer from similar diseases to non-immigrants, but their risks of getting diseases and their access to treatments and healthcare are different [37]. The sharp increase in cases in Germany (2015) and Sweden (2005–2006, 2017) could be a result of the growing number of involuntary immigrants in these countries. An Italian study also emphasized the significance of the mothers’ nationality; a multivariate model showed that it has an impact on the children’s caries experience in primary dentition [38]. In EU member states, immigrants may use/have less access to preventive services, including dental checkups and screenings, and these differences are especially perceptible in the younger population [39].

During recovery after the 2008 economic crisis, GDP per capita rose above or returned to its 2008 level by 2013 in most regions in the North (except Finland), West, and East (except Croatia and Slovenia) of the EU. However, Southern EU countries were hit harder by the crisis, especially Greece, Italy, Spain, Cyprus, and Portugal, and GDP was still below its 2008 level by 2014; thus, the recovery was not equally achieved in the EU [40]. Our oral disease burden findings are in line with this, as there is no observable uniformity regarding the changes in YLDs rates during or after recovery. According to our findings, there is a reverse significant association between GDP and urbanization and ECC burden, which indicates that greater economic development may contribute to the reduction in ECC rate. Thus, actions resulting in GDP growth and reduced inequality between urban and rural areas can lower YLDs rate. GDP, as a proxy of the living conditions of individuals, would be considered as one of the major determinants of oral health. Programs/actions increasing GDP (PPS) per capita are expected to effectively contribute to the reduction in the disease burden at national level, especially in Eastern countries of Europe. This contradicts the results of a recent association study, where better ecosystem vitality was associated with lower ECC risk, through the rational use of resources, healthy lifestyles, and preventive health practices [41]. However, lower incidence in Western countries is unsurprising, as the stronger the economic force, the more funds allocated to healthcare, which may facilitate the provision of preventive services [42]. In our analysis, we failed to detect a significant independent association between risk of poverty rate and YLDs. The fact that there is no clear link between the ECC burden and poverty rate might be explained by the disadvantaged/vulnerable groups of society that do not benefit from increased economic performance. Additionally, in most European countries, free access to oral health care services for children is funded by the public health system [43]. Thus, our results indicate that GDP per capita is a more useful indicator than risk of poverty rate in determining the oral health in population. Nevertheless, GDP and urbanization have to be taken into consideration as possible factors that affect population health [41].

According to the growing body of literature, the increasing ECC rate is associated with higher sugar intake [44]. In a worldwide ecological study, the per capita added sugar consumption in low-, middle-, and high-income countries was assessed. The added sugar consumption in low-income countries was found to be significantly lower than in middle- and high-income countries. However, this study found a correlation between the prevalence of ECC and sugar consumption only in middle-income countries [44]. The multivariate analysis revealed that the contribution of sugar consumption at population level has a significant impact on YLD, which suggests that YLD could be improved by lowering sugar consumption. Children and adolescents in Europe tend to consume more sugar-sweetened food than the current recommendations [45]. Furthermore, a review paper reported that, in the 10 examined European countries, children are more prone to consuming sweetened products than adults, and there is a trend that they consume more added sugars [46]. It also emphasized that there is a general association between a carbohydrate-based diet and socioeconomic factors; thus, the link between caries and socioeconomic factors, including education level, is very complex. The dose–response relationship between sugar consumption and ECC may explain the increasing ECC burden [44,47], primarily in less-privileged societies of the EU [48], and there is also a significant association with education level. Children of less educated mothers tend to consume more sugar in their diet, or be exposed to poor nutrition, and are less likely to visit the dentist [25,49,50,51,52]. The role of sugar consumption may also overwhelm preventive measures of routine dental checkups or other preventive factors [44].

Healthcare and preventive measures are mainly regulated on a national level in the EU. Socioeconomic factors with a long-term effect on health, including health status, risk factors, and access to healthcare, are mainly tackled by individual countries. There is a need for better understanding how socioeconomic and behavioral factors influence ECC, and more effort is necessary to create effective public health programs targeting the highest-risk children. A previous GBD (2017) study also emphasized that dental services should be a part of the universal healthcare system, and caries prevention is essential to the successful handling of the disease burden [32].

Dental health professionals are considered to be in a position to detect caries and address problematic sugar-related behaviors in children. Additionally, they play a role in advocacy by bringing the stakeholders together and facilitating the implementation of effective policy tools including taxes, warning labels, and policy changes that can help to reduce added sugar intake, prevent tooth decay, and improve health outcomes in vulnerable child populations [21]. The right feeding practices are important for children at an early age; thus, effective prevention may include parents beyond the dental team [53]. Pediatric primary care providers can also facilitate detecting the disease early and refer patients to pediatric dentists if necessary [54].

### Strengths and Limitations

Using the knowledge available, this study provided unique data on patterns and trends of burden due to ECC in the EU and analyzed the association with socioeconomic factors and sugar consumption. One of the limitations of the study is the interval covered; a relatively short period, between 2014 and 2017, was analyzed due to limited data availability. The relationship between the risk factors and socioeconomic aspects of ECC is complex, considering the number of different variants encompassed in the comparisons between countries. Careful interpretation of the results, based on aggregate data, is required as exposures may vary within countries, which is not accounted for in the analysis. Data may show huge variations in some countries due to the heterogeneity of data collection. Data availability highly limited the association models presented in the study. For example, incorporating the expenditure on caries prevention and provision of dental services into the analysis was not possible due to the lack of data. Out-of-pocket expenditure is a prominent part of the total oral healthcare-related expenditure in many member states, resulting in poor access to oral health services and consequential poor health outcomes. However, children and adolescents have access to free public preventive dental services in the member states of European Union [25]. Although there is evidence for the effects of the mother’s education level [13], mother’s nationality [38], access to dental care [55], and fluoride exposure [56], these indicators were not incorporated into our model due to insufficient/lack of longitudinal data. We also would like to highlight that the “under 5” age group used in this analysis does not fully correspond to the age group or age points used by ECC literature (see above), which may affect interpretation of our findings and impede their comparison with other literature data.

## 5. Conclusions

The burden due to caries of primary teeth among children under 5 years old shows unfavorable patterns and trends in the last 30 years. Unfortunately, systematic data on risk factors of ECC are limited, despite this being a prerequisite of successful caries control and prevention programs. Our study confirms that socioeconomic factors, including GDP per capita in PPS, urbanization, and sugar consumption, are associated with ECC burden. However, such an association is complex. Although several initiatives have been launched to tackle this chronic disease in most member states, we are lacking a systematic approach incorporating the management of socioeconomic factors related to health inequalities and excess sugar consumption, and improved data gathering. Our study indicates that more national efforts and EU policy actions are needed to change the current unfavorable situation.

## Figures and Tables

**Table 1:** Mean change in YLDs and covariates over time (2014–2019).

	2014–2019	2014	2015	2016	2017	2018	2019	Change over Time between 2014 and 2019		
	Mean (SD)	Mean (SD)	Mean (SD)	Mean (SD)	Mean (SD)	Mean (SD)	Mean (SD)	Absolute Difference	Relative Difference %	*p*-Value
YLDs rate	11.05 (4.86)	10.82 (5.11)	10.82 (5.09)	10.91 (5.02)	11.02 (4.99)	11.21 (4.81)	11.53 (4.51)	0.714	6.60%	0.582
Sugar consumption (kg/capita/year) *	54.92 (26.83)	54.3 (27.28)	53.9 (27.38)	55.37 (27.00)	56.11 (27.07)	-	-	1.812 *	3.34% *	0.801
At-risk-of-poverty rate (%)	16.72% (4.08)	16.85% (3.82)	17.07% (4.01)	17.05% (3.99)	16.8% (3.95)	16.79% (3.88)	15.74% (4.95)	−0.011	-6.59%	0.351
GDP per capita in PPS	101.11 (42.66)	99.57 (42.87)	101.14 (44.79)	100.82 (44.24)	101.18 (42.93)	101.82 (42.92)	102.11 (42.04)	2.536	2.55%	0.824
Urbanization (%)	38.78% (14.44)	38.54% (13.98)	38.34% (14.05)	39.02% (14.44)	39.06% (14.5)	39.91% (14.96)	37.82% (15.88)	−0.007	−1.86%	0.858
Number of observations	*N* = 168	*N* = 28								

* The change over time related to sugar consumption could be investigated between 2014 and 2017 due to the availability of data. Abbreviations: years lived with disability (YLDs), standard deviation (SD), number (N), gross domestic product (GDP), purchasing power standards (PPS).

**Table 2:** Panel data regression models for the effect of sugar consumption, at-risk-of-poverty rate, GDP per capita, and urbanization on YLDs rate of early childhood caries over time (2014–2017).

	Univariate Analysis			Multivariate Analysis *		
	Coefficient	95% CI	*p*-Value	Coefficient	95% CI	*p*-Value
Sugar consumption (kg/capita/year)	−0.016	−0.050, 0.018	0.364	0.104	0.072, 0.136	<0.001
At-risk-of-poverty rate (%)	0.270	0.038, 0.501	0.023	0.023	−0.146, 0.192	0.790
GDP per capita in PPS	−0.058	−0.077, −0.039	<0.001	−0.113	−0.134, −0.092	<0.001
Urbanization (%)	−0.100	−0.163, −0.037	0.002	−0.151	−0.195, −0.107	<0.001

* Significant results are shown in bold. Abbreviations: years lived with disability (YLDs), confidence interval (CI), gross domestic product (GDP), purchasing power standards (PPS).

## Data Availability

Three public databases were used for the analysis: Global Burden of Disease (GBD) database (Global Health Data Exchange), available online at http://ghdx.healthdata.org/gbd-results-tool. EUROSTAT database, available online at https://ec.europa.eu/eurostat/data/database. FAOSTAT database, available online at http://www.fao.org/faostat/en/#home.

## Supplementary Materials

The following are available online at https://www.mdpi.com/2072-6643/13/2/455/s1, Table S1: Incidence rate, prevalence, and YLDs rate among children aged under 5 years old and age-standardized YLDs rate of caries of primary teeth, 2019.
