# Supplementary material for: The Burden of Early Childhood Caries in Children under 5 Years Old in the European Union and Associated Risk Factors: An Ecological Study
Source: Nutrients. 2021 Jan 29;13(2):455. doi: 10.3390/nu13020455 (PMC7911369; doi:10.3390/nu13020455)
Supplement: Supplementary file 1 [file nutrients-13-00455-s001.pdf]

Table S1. Incidence rate, Prevalence, YLDs rate among children aged under 5 years old and Age-standardized YLDs Rate of caries of primary teeth, 2019

EU member state	Sex	Incidence rate (per 100,000 population) **(95% UI)**	Prevalence (%) **(95% UI)**	YLDs rate **(per 100, 000 population) (95% UI)**	Age-standardized YLDs rate **(per 100, 000 population) (95% UI)**
Austria	Male	39653,1	33,3%	7,8	2,1
	Female	38184,0	26,6%	7,2	2,0
	Both	38942,0	29,8%	7,5	2,0
Belgium	Male	40492,3	35,4%	8,1	2,1
	Female	38751,5	28,6%	7,7	2,1
	Both	39642,6	31,8%	7,9	2,1
Bulgaria	Male	51563,4	55,9%	16,8	3,3
	Female	51983,6	54,3%	17,0	3,3
	Both	51767,6	55,1%	16,9	3,3
Croatia	Male	51830,5	56,3%	16,7	3,3
	Female	52291,2	56,8%	16,9	3,3
	Both	52054,4	56,5%	16,8	3,3
Cyprus	Male	39115,1	31,6%	7,8	2,1
	Female	37673,9	25,2%	7,3	2,0
	Both	38419,1	28,3%	7,6	2,0
Czechia	Male	51888,2	56,9%	16,7	3,3
	Female	52038,2	56,0%	16,7	3,3
	Both	51961,3	56,5%	16,7	3,3
Denmark	Male	33944,3	25,1%	5,6	1,4
	Female	31965,2	19,3%	5,1	1,4
	Both	32981,1	22,0%	5,3	1,4

Estonia	Male	51034,3	56,8%	16,5	3,3
	Female	50990,9	54,5%	16,3	3,3
	Both	51013,2	55,6%	16,4	3,3
Finland	Male	43867,1	40,9%	10,0	2,4
	Female	41837,9	33,7%	9,1	2,3
	Both	42875,7	37,2%	9,6	2,4
France	Male	37956,1	34,0%	7,8	2,0
	Female	36876,2	28,2%	7,3	2,0
	Both	37428,1	31,0%	7,6	2,0
Germany	Male	40892,7	37,0%	8,8	2,1
	Female	37472,4	30,2%	8,1	1,9
	Both	39229,0	33,5%	8,5	2,0
Greece	Male	41687,1	41,3%	10,2	2,4
	Female	39548,7	33,1%	9,2	2,3
	Both	40643,6	37,0%	9,7	2,4
Hungary	Male	51765,9	56,5%	16,8	3,3
	Female	51893,8	55,4%	16,8	3,3
	Both	51828,1	55,9%	16,8	3,3
Ireland	Male	38046,3	30,0%	6,7	1,8
	Female	36555,3	24,2%	6,3	1,7
	Both	37319,2	26,9%	6,5	1,8
Italy	Male	45231,9	44,5%	11,9	2,6
	Female	43565,6	36,6%	11,0	2,5
	Both	44422,0	40,4%	11,5	2,5
Latvia	Male	51521,7	57,8%	16,6	3,3
	Female	51099,6	55,3%	16,4	3,3
	Both	51317,9	56,5%	16,5	3,3
Lithuania	Male	51173,6	57,8%	16,7	3,3
	Female	51077,6	55,0%	16,6	3,3
	Both	51126,9	56,4%	16,6	3,3

Luxembourg	Male	39743,8	34,4%	7,8	2,1
	Female	37730,3	26,8%	7,1	2,0
	Both	38760,1	30,4%	7,5	2,0
Malta	Male	39887,8	34,2%	8,0	2,1
	Female	37954,9	26,7%	7,2	2,0
	Both	38953,7	30,3%	7,6	2,0
Netherlands	Male	43888,0	36,5%	8,2	2,2
	Female	43285,5	29,8%	7,9	2,2
	Both	43594,3	33,0%	8,1	2,2
Poland	Male	53418,3	57,2%	16,8	3,2
	Female	53355,3	56,0%	16,7	3,2
	Both	53387,7	56,6%	16,7	3,2
Portugal	Male	39780,1	33,8%	7,9	2,1
	Female	38489,0	27,1%	7,4	2,0
	Both	39148,6	30,2%	7,7	2,1
Romania	Male	51848,0	55,4%	17,0	3,3
	Female	52452,2	54,8%	17,1	3,3
	Both	52141,6	55,1%	17,1	3,3
Slovakia	Male	51134,1	56,5%	16,8	3,3
	Female	51512,7	55,2%	16,7	3,3
	Both	51318,5	55,8%	16,7	3,3
Slovenia	Male	51933,6	57,0%	16,6	3,3
	Female	52041,3	56,1%	16,8	3,3
	Both	51986,0	56,5%	16,7	3,3
Spain	Male	41882,2	40,4%	10,3	2,4
	Female	38888,3	31,6%	8,2	2,1
	Both	40427,4	36,1%	9,2	2,3
Sweden	Male	45561,6	49,6%	12,8	2,7
	Female	43887,5	42,3%	12,3	2,7
	Both	44749,4	45,9%	12,5	2,7

United Kingdom	Male	16247,5	21,8%	4,9	1,4
	Female	15261,3	16,8%	4,5	1,3
	Both	15766,8	19,1%	4,7	1,3
European Union	Male	39535,01	40.33%	10,05	2,30
	Female	38061,91	34.24%	9,47	2,22
	Both	38817,68	37.20%	9,77	2.26

UI: uncertainty interval. Health indicators as defined by the GBD are as follows: incidence, meaning the number of new cases of a given cause during a given period in a specified population; prevalence, which is the proportion of people in a population who comprise a case of a disease, injury, or sequela; and YLDs (Years lived with disability), which means the years lived with any short-term or long-term health loss, weighted for severity by the disability weights. Data source is the Global Burden of Disease Collaborative Network. Seattle, Institute for Health Metrics and Evaluation (IHME), 2021. Available from http://ghdx.healthdata.org/gbd-results-tool
